# The C57BL/6J Mouse Strain Background Modifies the Effect of a Mutation in *Bcl2l2*

**DOI:** 10.1534/g3.111.000778

**Published:** 2012-01-01

**Authors:** Stefanie J. Navarro, Tuyen Trinh, Charlotte A. Lucas, Andrea J. Ross, Katrina G. Waymire, Grant R. MacGregor

**Affiliations:** Department of Developmental and Cell Biology, School of Biological Sciences, and Center for Molecular and Mitochondrial Medicine and Genetics, University of California Irvine, Irvine, California 92697-2300

**Keywords:** –*Nnt*, mutation, genetic modifier, BCL-W, apoptosis

## Abstract

*Bcl2l2* encodes BCL-W, an antiapoptotic member of the BCL-2 family of proteins. Intercross of *Bcl2l2* +/− mice on a mixed C57BL/6J, 129S5 background produces *Bcl2l2* −/− animals with the expected frequency. In contrast, intercross of *Bcl2l2* +/− mice on a congenic C57BL/6J background produces relatively few live-born *Bcl2l2* −/− animals. Genetic modifiers alter the effect of a mutation. C57BL/6J mice (*Mus musculus*) have a mutant allele of *nicotinamide nucleotide transhydrogenase* (*Nnt*) that can act as a modifier. Loss of NNT decreases the concentration of reduced nicotinamide adenine dinucleotide phosphate within the mitochondrial matrix. Nicotinamide adenine dinucleotide phosphate is a cofactor for glutathione reductase, which regenerates reduced glutathione, an important antioxidant. Thus, loss of NNT activity is associated with increased mitochondrial oxidative damage and cellular stress. To determine whether loss of *Bcl2l2 −/−* mice on the C57BL/6J background was mediated by the *Nnt* mutation, we outcrossed *Bcl2l2* congenic C57BL/6J (*Nnt −/−*) mice with the closely related C57BL/6JEiJ (*Nnt +/+*) strain to produce *Bcl2l2 +/− ; Nnt +/+* and *Bcl2l2 +/− ; Nnt −/−* animals. Intercross of *Bcl2l2 +/− ; Nnt +/+* mice produced *Bcl2l2 −/−* with the expected frequency, whereas intercross of *Bcl2l2 +/− ; Nnt −/−* animals did not. This finding indicates the C57BL/6J strain background, and possibly the *Nnt* mutation, modifies the *Bcl2l2* mutant phenotype. This and previous reports highlight the importance of knowing the genetic composition of mouse strains used in research studies as well as the accurate reporting of mouse strains in the scientific literature.

Inbred C57BL/6J mice are widely used in biological and biomedical research and for this reason the strain was selected to be the first mouse genome sequenced by a public consortium (Waterston *et al.* 2002). The use of inbred mouse strains that are presumed to be genetically homogenous at all loci reduces variability during analysis of a defined genetic modification, increases experimental reproducibility between different laboratories, and facilitates genetic mapping of strain-specific effects. Genetic drift is a concern in maintaining inbred species, and programs have recently been developed to monitor the genetic status of commonly used inbred mouse strains at commercial breeding facilities (Taft *et al.* 2006). Before such monitoring, C57BL/6J mice in some production facilities developed significant genetic alterations. For example, both the *Snca* gene, which encodes alpha-synuclein, and the adjacent *multimerin1* locus were mutated by a 365-kb deletion that arose spontaneously in the C57BL/6JOlaHsd strain in England some time before 1999 (Specht and Schoepfer 2001). Similarly, the C57BL/6J substrain of C57BL/6 has a deletion of 17.8 kb of the *Nnt* gene, which encodes nicotinamide nucleotide transhydrogenase (Huang *et al.* 2006). The *Nnt^C57BL/6J^* mutant allele arose spontaneously at The Jackson Laboratory in Bar Harbor, Maine, between 1976 and 1984. The allele has an in-frame deletion of exons 7−11 and a missense (M35T) mutation in the mitochondrial leader peptide sequence that results in reduced expression of *Nnt* mRNA and no functional NNT protein (Huang *et al.* 2006).

NNT is located in the inner mitochondrial membrane, where it functions as a redox-dependent proton pump that uses the proton gradient across the inner mitochondrial membrane to catalyze interconversion of nicotinamide adenine dinucleotide phosphate (NADPH) and NAD^+^ from NADP^+^ and NADH in the mitochondrial matrix (Earle *et al.* 1978; Pedersen *et al.* 2008). NNT activity has been estimated to account for approximately 45% of total production of NADPH, with the remainder coming from the pentose phosphate pathway, mitochondrial NAD(P)-malic enzyme, and NADP-isocitrate dehydrogenase (Sauer *et al.* 2004; Vogel *et al.* 1999). In the mitochondrial matrix, NADPH is a cofactor for glutathione reductase, which catalyzes conversion of oxidized glutathione disulfide to glutathione (GSH) (Dalton *et al.* 2004; Vogel *et al.* 1999). Replenishment of this antioxidant (GSH) is important to control reactive oxygen species (ROS) and cellular redox status (Dalton *et al.* 2004). Loss of NNT activity is associated with decreased NADPH, which in turn reduces the ratio of GSH/glutathione disulfide, thereby making the mitochondrial environment more susceptible to ROS-induced damage (Arkblad *et al.* 2005; Sheeran *et al.* 2010).

Hence, a prediction is that the *Nnt* mutation in C57BL/6J would render mice more sensitive to genetic or environmental factors that influence cellular stress. Indeed, the *Nnt* mutation acts as a genetic modifier, causing mice lacking the mitochondrial matrix localized superoxide dismutase 2 to display a more severe phenotype in which they die during embryogenesis (Huang *et al.* 2006; Kim *et al.* 2010). Here we report the effect of introducing a mutation of *Nnt* on the phenotype of *Bcl2l2 −/−* mice that lack a death-protecting member of the BCL-2 family of proteins.

BCL-2 proteins play a central role in controlling apoptosis (Cory and Adams 2002; Danial and Korsmeyer 2004; Taylor *et al.* 2008). Previously, we generated mice mutant for *Bcl2l2*, which encodes BCL-W, an antiapoptotic member of the BCL-2 family of proteins (Ross *et al.* 1998). Intercross of *Bcl2l2* +/− mice on either mixed 129B6 (Ross *et al.* 1998) or 129, FVB strain background (Print *et al.* 1998) produced *Bcl2l2* −/− mice with the expected frequency. In contrast, we show here that most *Bcl2l2 −/−* mice on a congenic C57BL/6J (*Nnt* mutant) background die before or at birth. We predicted that mutation of *Nnt* in C57BL/6J mice modifies the *Bcl2l2* mutant phenotype. To test this prediction, we introduced a wild-type allele of *Nnt* by outcrossing *Bcl2l2* mutant mice on a C57BL/6J (*Nnt −/−*) congenic background with the closely related C57BL/6JEiJ (*Nnt +/+*) strain, followed by intercrossing and genetic analysis of the ratio of *Bcl2l2 −/−* offspring recovered on either a *Nnt −/−* or *Nnt +/+* background. The results indicate that *Bcl2l2 −/−*; *Nnt* *+/+* mice are born alive at the expected frequency whereas *Bcl2l2 −/−*; *Nnt −/−* mice are not. Hence, the mutated *Nnt* allele, or a closely linked mutation, in C57BL/6J mice acts as a modifier of the mutant phenotype of loss of *Bcl2l2*.

## Materials and Methods

### Mice

C57BL/6J (*Nnt −/−*; cat. no. 000664) were purchased from The Jackson Laboratory (Bar Harbor, ME) in 1993 and have been maintained in our laboratory since then as a closed colony. The C57BL/6JEiJ (*Nnt +/+*, cat. no. 000924) substrain of C57BL/6J was established by Dr. Eva Eicher in 1976 at F129. Male C57BL/6JEiJ mice were purchased from The Jackson Laboratory in 2007 at F129+75. The ROSA41 strain has a mutant allele of *Bcl2l2*, encoding BCL-W (Ross *et al.* 1998). The mutation is generated by an insertion of the ROSA β-gal gene trap vector (Friedrich and Soriano 1991). The mutant allele is null for *Bcl2l2* function (Ross *et al.* 1998). The ROSA41 mutation was initially produced on a mixed 129S5, C57BL/6J strain background (Friedrich and Soriano 1991; Ross *et al.* 1998), but has since (as mentioned in the section *Crosses*) been backcrossed to a congenic C57BL/6J background. Mice on this strain background are available from the Mutant Mouse Regional Resource Center (UC Davis). Mice were provided Purina 5020 and nonacidified tap water *ad libitum*. Mice were housed in Techniplast ventilated cages with Bed O’Cobs bedding (The Andersons, Inc, Maumee, OH) and cotton nestlets for enrichment, with a light cycle of 13-hr on, 11-hr off, with lights on at 0630. Experiments involving animals were approved by the UCI Institutional Animal Care and Use Committee.

### Crosses

B6129-*Bcl2l2^GtROSA41Sor^ +/−* were backcrossed for 12 generations with C57BL/6J mice. To ensure that the Y chromosome and mitochondrial DNA were of C57BL/6J origin, at least one backcross was performed with a male or female C57BL/6J mouse respectively. N12Fx mice were intercrossed to generate the data in Table 1.

**Table 1:** Genotype of *Bcl2l2* mice recovered from intercross of *Bcl2l2^GtROSA41Sor^ +/−* on N12Fx C57BL/6J congenic (*Nnt −/−*) and [C57BL/6J × C57BL/6JEiJ ] F2 (*Nnt +/+* or *Nnt −/−*) strain background

*Bcl2l2^GtROSA41Sor^*					
Strain Background	+/+	+/−	−/−	Total	
N12Fx C57BL/6J.129S5 (*Nnt −/−*) LIVE (% total)	115 (30.3%)	208 (54.9%)	56 (14.8%)	379 (100%)	χ^2^ 21.98; 2 df; *P* = 6.35 E^−08^
N12Fx C57BL/6J.129S5 (*Nnt −/−*) DEAD (% total)	41 (24.1%)	66 (38.8%)	63 (37.1%)	170 (100%)	χ^2^ 14.19; 2 df; *P* = 0.00083
[C57BL/6J x C57BL/6JEiJ] F3 (*Nnt +/+*) (% total)	29 (26.4%)	53 (48.2%)	28 (25.5%)	110 (100%)	Fisher’s exact test, two-tailed *P* = 0.0014*^a^*
[C57BL/6J x C57BL/6JEiJ] F3 (*Nnt −/−*) (% total)	25 (34.3%)	43 (58.9%)	5 (6.8%)	73 (100%)		

a Respective χ^2^ goodness-of-fit analysis values (*Nnt −/−*; χ^2^ = 13.27; *2 df*; *P* = 0.0013, *Nnt +/+* χ^2^ = 0.164; *2 df*; *P* = 0.921). Fisher’s exact test, two-tailed, was calculated by generating a 2×2 contingency table using the genotype of *Nnt* (+/+ or −/−) and the *Bcl2l2* genotype (*+/+* combined with *+/−* or *−/−*).

The wild-type allele of *Nnt* was introduced by outcrossing *Bcl2l2^GtROSA41Sor^ +/−; Nnt ^C57BL/6J^ −/−* females on a C57BL/6J congenic background with male C57BL/6JEiJ mice. F1 animals were genotyped for both *Bcl2l2* and *Nnt*, and *Bcl2l2^GtROSA41Sor^ +/−* ; *Nnt +/−* animals were intercrossed. F2 animals were genotyped as before and *Bcl2l2^GtROSA41Sor^ +/−* ; *Nnt +/+*, and *Bcl2l2^GtROSA41Sor^ +/−* ; *Nnt −/−* animals were selected to establish breeding pairs for intercross. Genotypes in Table 1 were recorded from F3 or F4 crosses. *Bcl2l2* (chromosome 14) and *Nnt* (chromosome 13) segregate independently.

### Genotyping by semiquantitative polymerase chain reaction analysis of *Nnt* or *Bcl2l2* locus

C57BL/6J and C57BL/6JEiJ strains were genotyped for their respective *Nnt* alleles by the use of a three-primer, two-allele polymerase chain reaction assay that discriminates between the wild-type allele of *Nnt* in C57BL/6JEiJ and the mutant allele lacking exons 7−11 in C57BL/6J mice as described (Nicholson *et al.* 2010). Primers were designed by use of the publically available mouse genome sequence of the *Nnt* locus (Ensembl). The “COM” primer (5′-GTAGGGCCAACTGTTTCTGCATGA-3′) participates in amplification of both the wild-type and *Nnt^C57BL/6J^* mutant alleles, whereas the “WT” (5′-GGGCATAGGAAGCAAATACCAAGTTG-3′) and “MUT” (5′-GTGGAATTCCGCTGAGAGAACTCTT-3′) primers are specific to the wild-type and *Nnt^C57BL/6J^* mutant alleles, respectively. The amplification products are 579 bp for the wild-type allele and 743 bp for the mutant allele. Use of the primers with heterozygous mutant *Nnt^C57BL/6J^* template produces an additional faint product of approximately 900 bp that assists assignment of genotype. Amplification was performed in 25 μL with the use of 1x Go-Taq Flexi (Promega, Madison, WI) buffer with final concentration of 2.5 mM MgCl_2_, 1.0 μM Nnt-COM, 0.33 μM Nnt-WT, 0.67 μM Nnt-MUT, 0.67 mM dNTPs, and 1.25 units Go-Taq Flexi enzyme (Promega). Amplification conditions used were initial melt 95°, 5 min; then 35 cycles of 95°, 45 sec; 58°, 30 sec, 72°, 45 sec; followed by a final extension of 5 min at 72°. Mice containing the *Bcl2l2^GtROSA41Sor^* mutant allele were genotyped as described (Ross *et al.* 1998).

### Statistical analysis

Differences between the expected and observed frequencies of animals of different genotypes were analyzed with a χ^2^ goodness-of-fit-test, and two-tailed Fisher’s exact test via the method of summing small *P* values (Graph Pad Software, La Jolla, CA). Significance was defined as *P* ≤ 0.05.

## Results and Discussion

Intercross of *Bcl2l2^GtROSA41Sor^ +/−* on a mixed C57BL/6J, 129S5 strain background produced *Bcl2l2 −/−* mice with the expected frequency (Ross *et al.* 1998). An independent laboratory reported the same result from intercross of *Bcl2l2 +/−* mice on a mixed 129S1, C57BL/6J, FVB strain background (Print *et al.* 1998). In contrast, after backcross to a congenic C57BL/6J background, homozygous mutant *Bcl2l2^GtROSA41Sor^* mice were recovered at a significantly reduced frequency (Table 1). Genotyping of dead animals found among newborn offspring revealed a significant increase in homozygous mutant animals (Table 1). Visual inspection, necropsy, and histology of dead animals provided no obvious insight into the cause of death of *Bcl2l2 −/−* animals (data not shown).

To determine whether mutation of *Nnt*, or a closely linked locus, in the C57BL/6J strain caused the reduced frequency of live *Bcl2l2 −/−* animals, we restored *Nnt* function by outcrossing congenic C57BL/6J *Bcl2l2^GtROSA41Sor^ +/−* N12Fx female mice with C57BL/6JEiJ (*Nnt +/+*) males. The C57BL/6JEiJ strain was selected because it is most closely related to C57BL/6J while being wild-type for *Nnt* (Huang *et al.* 2006; Petkov *et al.* 2004). To maintain the mitochondrial genotype of the congenic C57BL/6J *Bcl2l2^GtROSA41Sor^* animals, females were crossed with C57BL/6JEiJ males. Live *Bcl2l2 ^GtROSA41Sor^ −/−* offspring were recovered from intercross of [C57BL/6J × C57BL/6JEiJ] F2 *Bcl2l2^GtROSA41Sor^ +/−* ; *Nnt +/+* animals at the expected frequency (Table 1). In contrast, *Bcl2l2* −/− animals were recovered at a significantly reduced frequency from intercross of [C57BL/6J × C57BL/6JEiJ] F2 *Bcl2l2^GtROSA41Sor^ +/−*, *Nnt−/−* F2 animals (Table 1). This finding indicates that the C57BL/6J strain background modifies the phenotype of the *Bcl2l2 ^GtROSA41Sor^* mutation.

Our data implicate the *Nnt* mutation in C57BL/6J mice as the genetic modifier responsible for loss of a proportion of *Bcl2l2 −/−* animals. Mutation of *Nnt* increases the sensitivity of an animal to oxidative stress (Arkblad *et al.* 2005). Loss of BCL-W increases the likelihood that a cell (*e.g.* neurons, intestinal epithelial cells) will undergo apoptosis in response to cellular damage or oxidative stress (Courchesne *et al.* 2011; Pazyra-Murphy *et al.* 2009; Pritchard *et al.* 2000). Taken together, this suggests a model in which loss of NNT activity reduces the survival of BCL-W-deficient mice by increasing oxidative stress within cells in which BCL-W is normally expressed and that are essential for embryogenesis or early postnatal survival. However, whether the death of *Bcl2l2* −/−, *Nnt* −/− mice is caused by increased apoptosis of a specific cell type remains unknown. At present we cannot exclude the possibility that other loci on mouse chromosome 13 linked to *Nnt* contribute to this effect. However, on the basis of the aforementioned model, it seems plausible that the *Nnt* mutation in C57BL/6J mice contributes at least some, if not all, of the modifier effect that enhances the impact of loss of *Bcl2l2*.

The C57BL/6J strain background also modifies the severity of the phenotype of mice lacking manganese super-oxide dismutase 2 (*sod2*) (Huang *et al.* 2006; Kim *et al.* 2010). *Sod2 −/−* mice on a DBA/2J strain background are born at normal frequency but die at approximately postnatal day 8 as the result of severe acidosis. In contrast, most *Sod2 −/−* on a C57BL/6J background die at approximately embryonic day 15 as the result of dilated cardiomyopathy. A QTL analysis of long-lived *Sod2 −/−* mice generated from DBA/2J-*Sod2 +/−* mice back-crossed to C57BL/6J (N7) mapped the modifier locus to a 10-Mb region on distal chromosome 13, which contains *Nnt* (Huang *et al.* 2006). Mutation of the *Nnt* locus in C57BL/6J mice has also been shown to be responsible for glucose intolerance and defective insulin secretion in C57BL/6J mice (Freeman *et al.* 2006).

These results reemphasize the importance of knowing the genetic composition of mouse strains used in studies, as well as accurately reporting mouse strains in the scientific literature (Stevens *et al.* 2007; Taft *et al.* 2006; Wotjak 2003). They also emphasize the particular importance of knowing the substrain of C57BL/6 mice used in studies involving analyzing of cellular stress and cell death. The latter point is illustrated by a recent study (Ni *et al.* 2008), which confirmed a report (Bouillet *et al.* 2001) of increased severity of a mutant phenotype of *Bcl2 −/−* mice on a C57BL/6 background. Ni *et al.* 2008 described the C57BL/6 mice used as being from The Jackson Laboratory, although the authors did not specify which of the six sublines of C57BL/6 mice available at The Jackson Laboratory they used. The absence of such information hinders the present and future scientific community from fully interpreting a published report.

The advent of high-density single nucleotide polymorphism (SNP) genotyping platforms offers a powerful tool to monitor genetic stability within an individual sub-strain in a breeding facility (Petkov *et al.* 2004; Yang *et al.* 2009). Recently SNP mapping was used to identify a copy-number variation allele on chromosome 19 of C57BL/6J mice that affects expression of the *Ide* and *Fgfbp3* genes and that is segregating in what were presumed to be inbred C57BL/6J mice (Watkins-Chow and Pavan 2008). Continued improvements in methods and reduced cost of SNP genotyping should ultimately facilitate routine genotyping of mouse strains used in research studies.
